# Prevalence and causes of visual impairment and rate of wearing spectacles in schools for children of migrant workers in Shanghai, China

**DOI:** 10.1186/1471-2458-14-1312

**Published:** 2014-12-22

**Authors:** Jiangnan He, Lina Lu, Haidong Zou, Xiangui He, Qiangqiang Li, Weijie Wang, Jianfeng Zhu

**Affiliations:** Shanghai Eye Disease Prevention and Treatment Center, No. 380, Kangding Road, Jingan, Shanghai 20040 China; Center of Disease Control and Prevention of Baoshan District, No.158, Yueming Road, Baoshan, Shanghai 201901 China; Center of Disease Control and Prevention of Minhang District, No.965, Zhongyi Road, Minhang, Shanghai 201101 China

## Abstract

**Background:**

To assess the prevalence of visual impairment and rate of wearing spectacles in schools for children of migrant workers in Shanghai, China.

**Methods:**

Children from grade 1 to 5 in schools for children of migrant workers were randomly chosen for ocular examinations. All children were screened for uncorrected visual acuity and presenting visual acuity. After screening, the children whose uncorrected visual acuity was 20/40 or less received ocular motility evaluation, cycloplegic refraction/non-cycloplegic refraction, and external eye, anterior segment, media, and fundus examinations.

**Results:**

A total of 9673 children were enumerated and 9512 (98.34%) participated in this study. The prevalence of uncorrected, presenting, and best-corrected visual acuity of 20/40 or worse in the better eye were 13.33%, 11.26%, and 0.63%, respectively. The rate of wearing spectacles of the children with visual impairment in one or both eyes was 15.50%. Of these, 26.05% were wearing spectacles with inaccurate prescriptions. Refractive error was a major cause of visual impairment, accounting for 89.48% of all the visual impairment causes. Other causes of visual impairment included amblyopia accounting for 10.12%; congenital cataract, 0.1%; congenital nystagmus, 0.1%; ocular prosthesis, 0.1%; macular degeneration, 0.05%; and opaque cornea, 0.05%.

**Conclusions:**

This is the first study of the prevalence and causes of visual impairment in schools for children of migrant workers in Shanghai, China. The visual impairment rate in schools for children of migrant workers in suburbs of Shanghai in the best eye before vision correction was lower than those of urban children in mainstream schools in Guangzhou in 2012, and higher than students in rural of Beijing in 1998 and in suburb of Chongqing in 2007. The refractive error was the principal cause of the visual impairment of the children of migrant workers. The rate of wearing spectacles was low and the percentage of inaccurate prescriptions, among those who wore spectacles, was high. Uncorrected refractive error was a significant cause of visual impairment in migrant children.

## Background

Visual impairment in children is a severe public health, social, and economic problem worldwide [1–4]. Uncorrected refractive error, which is a remediable cause of visual impairment [5, 6], is one of the five priorities of the global initiative for elimination of avoidable visual disability. Accurate information on the prevalence and causes of visual impairment in children may help health organizations prioritize resources and develop appropriate policies on human resources and infrastructure. Such information may also facilitate development of screening programs to identify people at an increased risk for eye diseases.

Eight Refractive Error Studies in Children (RESC) were conducted in Nepal [7], China [8, 9], Chile [10], India [11, 12], South Africa [13], and Malaysia [14] in recent years. These studies examined the associations between refractive error and visual impairment. All of these studies emphasized the finding that uncorrected refractive error is one of the most common causes of visual impairment. However, these studies were mainly conducted in urban and rural populations. In recent years, millions of rural-to-urban migrants have moved to large cities like Shanghai to seek opportunities. According to the data from the National Population and Family Planning Commission (NPFPC) of the Government of the People’s Republic of China, the migrant population reached 230 million in 2011, accounting for 17% of the total national population [15]. More and more migrant workers move with their families or start families after having arrived in the cities, which has increased the number of school-aged migrant children in cities. Unfortunately, most migrant children cannot register for public services in cities, such as health and education, because they are still registered as rural residents and are officially expected to receive these services in their places of registration. As a consequence, millions of migrant children only have access to poor-quality healthcare services and attend low-quality schools like migrant children’s schools [16]. Given the high prevalence of uncorrected vision among children in China, including (perhaps especially) children of migrant workers, it is important to determine the prevalence and causes of visual impairment and rate of wearing spectacles. Uncorrected visual impairment has a negative impact on academic achievement because of its effects on sensory perception, cognition, and school connectedness [17]. A positive change can be made in a large number of children of migrant workers who are at risk of falling behind their peers. Currently, there are no data on visual impairment in children of migrant workers, even though they number in the hundreds of millions. This paper reports the findings of visual impairment and rate of wearing spectacles among migrant workers' children, conducted in metropolitan Shanghai of China.

## Methods

### Sample selection

Shanghai is the economic, cultural, and scientific center of eastern China. According to the 2010 census, it had a resident population of 23.01 million, of which 39% were from the migrant population that had taken up residence in Shanghai [18]. Data from the Shanghai Education Commission of 2012 showed that Shanghai had 155 special migrant children’s schools, which enrolled a total of approximately 150,000 students. Among the 155 schools, 95% were primary schools, and they were mainly distributed in 9 districts and 1 county within Shanghai. (There are 16 districts and 1 county in Shanghai, among which 8 are downtown districts). Two-stage random sampling was used in this study. In the first stage, two suburban districts densely populated with migrant workers in the northern and southern parts of the Shanghai, Baoshan, and Minhang districts were randomly selected. In the second stage, a cluster sample of 7 schools was selected from the 27 schools for the children of migrant workers in Baoshan district, and 4 were selected from the 26 schools in Minhang district.

### Field operations

The present study adhered to the provisions of the Declaration of Helsinki for research involving human subjects. This study was approved by the Ethics Committees of Shanghai Eye Disease Prevention and Treatment Center. Shanghai Municipal Education Commission, Baoshan Bureau of Education, and Minhang Bureau of Education supported this study by sending notices to the selected schools instructing close cooperation.

Between February and March of 2013, Minhang and Baoshan Centers for Disease Control and Prevention (CDCs) contacted the authority in each of the selected schools to explain the project’s objectives and procedures. Each CDC obtained a list of the registered students, including name, age, and sex. Children, aged 7 to 12 years, were included in the enumerated sample .Children who were absent from the school at the time of the enumeration, due to illness or unauthorized absence, were excluded.

School authorities contacted the parents and guardians of all the children to obtain formal consent for cycloplegic refraction. The project staff organized prescheduled meetings with parents, guardians, teachers, and students to explain the details of eye examinations. Examinations were conducted during school hours, mainly between April 23 and June 6, 2013. The school teachers reminded the children who had no written consent before the examination date to bring a signed consent form to the school. Children without signed consent forms did not undergo cycloplegia, and instead underwent non-cycloplegic refraction and clinical ocular examination. Children who usually wore glasses were reminded to wear them on the day of the examination.

### Clinical examination procedures

Eye examination was performed between April 23 and June 6 of 2013 by a clinical team composed of 5 optometrists, 2 public health doctors, an ophthalmologist, a field assistant, a school nurse, and a study manager. All children received visual screening. Visual acuity was measured at 5 m by an ophthalmic technologist using a retroilluminated standard logarithmic visual acuity chart with tumbling-E optotypes. Visual acuity was measured both with and without glasses in children that wore glasses.

After visual screening, children with unaided (uncorrected) visual acuity of 20/40 or worse in either eye received ocular motility measurement and examination of the external eye, anterior segment, and media, cycloplegic/non-cycloplegic refraction, subjective refraction, and fundus examinations.

Ocular motility was evaluated by cover tests for near and distance. Prism–cover tests were used for tropic component measurements. Tropia was categorized as esotropia, exotropia, or vertical deviation. An ophthalmologist evaluated the external eye and anterior segment (eyelid, conjunctiva, cornea, iris, and pupil) with a handheld slit lamp. Cycloplegia was induced with 5 drops of 0.5% compound tropicamide administered 5 minutes apart. Cycloplegia and pupil dilation were then evaluated 20 minutes later after instillation of the last drop. Pupillary dilation of 6 mm or more without light reflex was considered to be complete cycloplegia. An ophthalmic technologist performed autorefraction with a refractor (auto-refractor KR-8900, Topcon, Tokyo, Japan). Subjective refraction was assigned to children for cycloplegic or non-cycloplegic refraction, and the best corrected visual acuity was recorded.

Children with visual vision of 20/40 or less in the worse eye were given prescription glasses free of charge to improve their vision by refractive correction. Students with amblyopia and low vision were transferred to the Shanghai Eye Disease Prevention and Treatment Center for further examination and treatment without charge. Students suffering from strabismus whose parents gave permission were also transferred to the center for surgery with their surgical expenses paid by Shanghai Children Charity Foundation.

### Data management and analysis

Data were entered with Epidata software (Epidata 3.0 TM) for Windows. All statistical analyses were conducted using SAS version 9.1.3 (SAS Institute, Gary, NC, U.S.A). Statistical significance was evaluated at *P* < 0.1 when confidence intervals were calculated by adjusting cluster effects associated with the sampling design. The effects of cluster design are represented by a ratio (termed, deff), which compares the estimate of actually obtained variance with the (generally smaller) variance that would have been obtained if simple random sampling had been used [19].

The prevalence of visual impairment with uncorrected (unaided), presenting, and best corrected visual acuity were calculated. The latter measurement was based on subjective refraction for the children with reduced uncorrected visual acuity. Children with unaided visual acuity of 20/40 or less in either eye were regarded as having visual impairment. Thresholds of 20/40 or less, 20/63 or less, and 20/200 or less were used to establish visual acuity categories.

Myopia was defined as spherical equivalent (SE) refractive error of at least −0.50 D and hyperopia as +2.00 D or more. Children were considered to have myopia or hyperopia if one or both eyes were myopic or hyperopic, respectively. Age-specific, sex-specific, and grade-specific prevalence rates of myopia and hyperopia were estimated for children with cycloplegia in both eyes.Astigmatism was investigated at cylinder values of 0.75 D or greater.

Amblyopia was considered the cause of impairment in eyes with best-corrected VA (BCVA) ≤20/40 and no apparent organic lesion if one or more of the following criteria were met: (1) esotropia, exotropia, or vertical tropia at 4-m fixation or exotropia or vertical tropia at 0.5 m. (2) anisometropia of 2.00 SE or more. (3)bilateral ametropia of at least +6.00 SE.

The rate of wearing spectacles refers to the percentage of children who were actually wearing spectacles in children with uncorrected visual acuity ≤20/40 in either eye.

## Results

### Study population

There were 9673 eligible students from 11 schools for children of migrant workers. A total of 9512 students (98.34%), 5508 boys and 4004 girls, underwent visual screening. Of these, 2985 students were 7–8 years old, 3495 aged 9–10, and 3032 aged 11–12. This did not differ significantly from the age distribution of the unexamined group (*P* = 0.0261). Of all the students examined in grades 1–5, 2274 were in the first grade, 2004 were in the second grade, 1721 were in the third grade, 1831 were in the fourth grade and 1682 were in the fifth grade. The grade distribution also did not differ significantly from the unexamined group (*P* = 0.0108). The detailed demographic characteristics of examined and unexamined objects are shown in Table 1.

**Table 1:** **Sex, age and grade distributions of the selected and examined population**

		Examined population	Not examined population	Enumerated population	***P***value
		(N, %)	(N, %)	(N, %)	
Sex	Male	5508 (57.91)	102 (63.35)	5610 (58.00)	0.1649
	Female	4004 (42.09)	59 (36.65)	4063 (42.00)	
Age (y)	7–8	2985 (31.38)*	40 (24.84)	3025 (31.27)	0.0261
	9-–10	3495 (36.74)	54 (33.54)	3549 (36.69)	
	11–12	3032 (31.88)^†^	67 (41.61)	3099 (32.04)	
Grade	1	2274 (23.91)	23 (14.29)	1857 (19.20)	0.0108
	2	2004 (21.07)	41 (25.47)	1722 (17.80)	
	3	1721 (18.09)	31 (19.25)	3025 (31.27)	
	4	1831 (19.25)	26 (16.15)	3549 (36.69)	
	5	1682 (17.68)	40 (24.84)	3099 (32.04)	
All		9512 (100.00)	161 (100.00)	9673 (100.00)	

*Five 6-year-old children were grouped with the 7–8-year olds.

^†^Ten 13-year-old children were grouped with the 11–12-year olds.

### Visual acuity

Table 2 shows the distribution of visual acuity. A total of 9512 students completed visual examination, 5508 were boys and 4004 girls. Of all the children examined, the uncorrected visual acuity in both eyes of 7506 (78.85%) children was 20/32 or better. The uncorrected visual acuity in the better eye was less than or equal to 20/40 in 1268 (13.33%) children, including 48 students (0.5%) with binocular blindness (visual acuity in the better eye less than 20/200).

**Table 2:** **Distribution of uncorrected, presenting, and best corrected visual acuity**

Visual acuity category	Uncorrected visual acuity	Wearing glasses*	Presenting visual acuity	Best visual acuity
	n (%; 95% CI) ^†^	n (%)	n (%; 95% CI) ^†^	n (%; 95% CI) ^†^
≥20/32 both eyes	7506 (78.91; 78.09–79.73)	17 (0.23)	7677 (80.71; 79.92–81.50)	9301 (97.78; 97.48–98.08)
≥20/32 one eye only	738 (7.75; 7.21–8.28)	44 (5.96)	764 (8.03; 7.48–8.58)	151 (1.59; 1.34-1.84)
≤20/40 to ≥ 20/63 better eye	887 (9.33; 8.75–9.92)	135 (15.22)	816 (8.58; 8.02–9.14)	43 (0.41; 0.21–0.78)^†^
≤20/80 to ≥ 20/160 better eye	333 (3.50; 3.13–3.87)	102 (30.63)	235 (2.34; 1.59–3.42)^†^	15 (0.14; 0.07 –0.31)^†^
≤20/200 better eye	48 (0.50; 0.33–0.75)^†^	30 (62.5)	20 (0.20; 0.11–0.38)^†^	2 (0.03; 0.004–0.19)^†^
All	9512 (100.00)	328 (3.45)	9512 (100.00)	9512 (100.00)

*The percentage within each visual acuity category is based on uncorrected vision.

^†^Confidence intervals (CIs) were calculated using the exact binomial distribution instead of the normal approximation, and cluster design effects were taken into account in calculating Cls for estimates based on the normal approximation ranging from 1.647 to 6.718.

A total of 328 children (3.45%) wore glasses for vision correction, among whom, 267 students had binocular uncorrected visual acuity of less than 20/40. The number of students, including those wearing glasses (presenting visual acuity) , with binocular visual acuity of less than 20/40 decreased to 1071 (11.26%). According to the results of subjective refraction testing (best corrected visual acuity), the number of students with visual impairment in both eyes decreased to 60 (0.63%), including 2 students (0.03%) suffering from binocular blindness.

The Kolmogorov-Smirnov test was used to assess the distribution of uncorrected, presenting, and best corrected visual acuity (*P* = 0.0248, *P* = 0.0281, and *P* = 0.6832) of the boys and girls separately. The uncorrected and presenting visual acuities of the boys was found to be better than in the girls, however there was no significant difference in the best corrected visual acuity between boys and girls.

### Spectacle wearing

Table 3 demonstrates the characteristics of the participants with visual impairment who were and were not wearing glasses. There were 311 students (15.50%) wearing glasses for correction of visual impairment, 167 are boys (53.7%) and 144 girls (46.30%). According to the results of univariate logistic regression analysis, the rate of spectacle wearing did not differ significantly by sex (*P* = 0.4959). As for the grade, the number of students wearing glasses increased from 27 in the first-grade to 119 in the fifth-grade. The result of the univariate logistic regression analysis also indicated that the difference between the numbers of students wearing glasses in the first grade and fifth-grade was significant. Regarding age group, there were 37 students (11.90%) who wore glasses within the age group of 7–8, which was less than the other groups. There were 108 (34.73%) in the 9–10 age group and 166 (53.38%) in the 11–12 age group. Univariate logistic regression analysis demonstrated that the number of students wearing glasses rose with age. With glasses, the presenting binocular visual acuity in 230 students (73.95%) was greater than 20/40, but remained below 20/40 in 81 students (26.05%).

**Table 3:** **Characteristics of 2006 visually impaired participants wearing and not wearing glasses**

		Wearing glasses	Not wearing glasses	P value	OR (95% CI)
		N (%)	N (%)		
Sex	Male	167 (53.7)	949 (55.99)		1
	Female	144 (46.30)	746 (44.01)	0.4959	1.10 (0.86,1.40)
Grade	1	27 (8.68)	211 (12.45)		1
	2	32 (10.29)	273 (16.11)	0.7514	0.92 (0.53,1.58)
	3	51 (16.40)	342 (20.18)	0.5462	1.17 (0.71,1.92)
	4	82 (26.37)	417 (24.60)	0.0703	1.54 (0.97,2.45)
	5	119 (38.26)	452 (26.67)	0.0016	2.06 (1.31,3.22)
Age (y)	7–8	37 (11.90)	316 (18.64)		1
	9–10	108 (34.73)	588 (34.69)	0.0265	1.57 (1.05,2.34)
	11–12	166 (53.38)	791 (46.67)	0.0026	1.79 (1.23,2.62)
All		311 (100.00)	1695 (100.00)		

### Cycloplegic dilation and refractive error

Cycloplegic dilation and refraction should have been performed in 2006 (21.09%) children based on an uncorrected visual acuity of 20/40 or worse in at least one eye. However, the parents of 810 (40.38%) children did not give consent for their children to undergo cycloplegic examination at school, so the cycloplegic rate (59.62%) of children with visual impairment was relatively lower due to the weak eye care consciousness of migrant children’s parents and deficiency of cycloplegic mobilization. Therefore, cycloplegic refraction was performed in a total of 1196 (59.62%) children. Pupillary dilation of at least 6 mm and absence of light reflex were achieved in 1195 (59.57%) right eyes, and absent light reflex without full dilation was achieved in 1 right eye. The respective numbers were 1183 (58.97%) and 13 (0.65%) in the left eyes.

Cycloplegic auto-refraction measurements were performed in 1196 children. Refractive error was measured for these children based on auto-refraction. The proportion of visual impairment with myopia was56.79 % to 43.21% across sex subgroups, 7.19% to 31.69% across grade subgroups, and14.07% to 48.13% across age subgroups (Table 4). The proportion of visual impairment with hyperopia was 50.00% to 50.00% across sex subgroups, 22.86% to 10.00% across grade subgroups, and 37.14% to 24.29% across age subgroups. The proportion of visual impairment with astigmatism was 54.98% to 45.02% across sex subgroups, 13.83% to 28.64% across grade subgroups, and 16.87% to 52.55% across age subgroups.

**Table 4:** The proportion of refractive error with visual impairment (one or both eyes) by sex, grade, and age*

	Myopia	Hyperopia	Astigmatism
	N (%)	N (%)	N (%)
Sex			
Male	577 (56.79)	35(50.00)	453(54.98)
Female	439 (43.21)	35(50.00)	371(45.02)
Grade			
1	73(7.19)	16(22.86)	114(13.83)
2	140(13.78)	18(25.71)	127(15.41)
3	200(19.69)	11(15.71)	162(19.66)
4	281(27.66)	18(25.71)	185(22.45)
5	322(31.69)	7(10.00)	236(28.64)
Age (y)			
7–8	143(14.07)	26(37.14)	139(16.87)
9–10	384(37.80)	27(38.57)	252(30.58)
11–12	489(48.13)	17(24.29)	433(52.55)
All	1016(100)	70 (100)	824(100)

*Refraction data were available for 1196 children with visual impairment performed cycloplegic auto-refraction measurements.

### Ocular abnormalities

A total of 2006 children with visual impairment have received ocular motility examinations, and 36 (1.79%) of them were obvious tropia. Among the children with tropia, 29 (80.56%) were male and 7 (19.44%) female. Among the 36 children with tropia, 28 (77.78%) children was present at both near and distance fixation, and 8 (22.22%) had both near and distant esotropia. With the near fixation, 61.11% of tropia was ≤ 30 prism diopters and with the distant fixation, 38.89%.

### Causes of visual impairment

Of the 2006 children with visual impairment, 1631 had visual impairment in the right eye, 1643 in the left eye, and 1268 in both eyes. The main cause of the visual impairment was refractive error. A total of 1795 (89.48%) among the 2006 children attained normal or near-normal acuity in both eyes with refractive correction (Table 5).

**Table 5:** Causes of visual impairment*

	Right eye	Left eye	Children with visual impairment	Prevalence in the population (%)
			n (%) (one or both eyes)*	(one or both eyes)*
Refractive error†	1478	1447	1795 (89.48)	18.87
Amblyopia	147	188	203 (10.12)	2.13
Ocular prosthesis	0	2	2 (0.10)	0.02
Congenital nystagmus	2	2	2 (0.10)	0.02
Congenital cataract	2	2	2 (0.10)	0.02
Macular degeneration	1	1	1 (0.05)	0.01
Opaque cornea	1	1	1 (0.05)	0.01
All	1631	1643	2006 (100.00)	

Data show the frequency (%) of cases.

*Children with visual acuity ≤ 20/40 in both eyes may represent two separate causes of reduced vision, one in each eye.

†Refractive error was assigned as the cause of reduced vision for all eyes correcting to ≥ 20/32 with subjective refraction, even if other contributing diseases were present.

Amblyopia, satisfying the predefined criteria, was the secondary cause of uncorrectable visual impairment in 203 (9.72%) children: 19 with tropia, 7 with hyperopia ≥ 6.00 SE diopters, and 169 with anisometropia ≥ 2.00 SE diopters. Other causes were found in 8 children: ocular prosthesis in two left eyes, congenital nystagmus, and congenital cataracts in two children each and macular degeneration and opaque cornea in one each.

## Discussion

This study was based on a charity research project jointly supported by Shanghai Charity Foundation and Shanghai Eye Disease Prevention and Treatment Center. It was also a pilot investigation on myopia, amblyopia, and low vision in all migrant workers' children’s schools in Shanghai, performed for the purpose of determining the prevalence and causes of visual impairment among the migrant children in Shanghai. The data collected in this study may facilitate future investigations in Shanghai or other areas where migrants congregate. This study, with the emphasis of refractive error and eye disease screening, is the first investigation on visual impairment for migrant workers' children.

The study showed that the visual impairment rate of the best eye before vision correction among children of migrant workers was 13.33%, but about with 91% of visual impairment was attributable to refractive error and mostly myopia. The visual impairment rate (13.33%) in schools for migrant children in suburbs of Shanghai was higher than in students aged 5–15 in rural areas in Shunyi District, Beijing (8.18%) and in students aged 6–15 in a suburb in Yongchuan County, Chongqing (7.69%) [8, 20]. Myopia is a common condition in urban areas of China, so children may suffer from myopia during the process of moving from country to city accompanied by more intensive schooling. In addition, migrant children maybe spend less time performing outdoor activities, which prevents the development of myopia, than children in rural areas where there is more open space and natural sunlight [21]. However, the children in Shanghai suburbs had a lower rate of visual impairment in the best eye before vision correction than those of urban children aged 5–15 in RESC Guangzhou (13.33% vs. 22.3%) [9]. In addition, compared with the prevalence of moderately and severely reduced visual acuity(visual acuity ≤20/32) in Guangzhou in 2002 [22], the uncorrected visual acuity in migrant children in Shanghai reduced slower from aged 7 group (19.20% vs. 19%), aged 8 group (20.14% vs. 21%) , aged 9 group (24.41% vs. 30%), aged 10 group (28.95% vs. 35%), aged 11 group (36.22% vs. 48%) to aged 12 group (41.83% vs. 60%). That may have resulted from the fact that 90% of migrant workers’ schools are not included in the Shanghai education system, which requires the students to study intensely [16]. The parents of migrant children maybe provide less extra tutoring due to limited economic capacity and poor educational consciousness. As a result, the children of migrant workers study less intensely, which prevents and slows the development of myopia, and leads to a lower rate of visual impairment. Further research will focus on the association study intensity of children of migrant workers with visual impairment.

Another result of this study was that, after correction, the visual acuity of 97.91% students reached or approached normal levels. For students with visual impairment in at least one eye, only 15.50% (311/2006) wore glasses, which was lower than aged 5-15 urban children in Guangzhou (15.50% vs.53.3%) and aged 5-15 rural children in Shunyi, Beijing (15.50% vs.20.55%) [8, 9]. A rural vision care project conducted in China revealed that although half of the children could benefit from spectacle wear, 62.3% were not wearing appropriate correction [23]. The major reason may be misinformation. A commonly-held (but mistaken) view is that wearing glasses is harmful to children’s eyes. If teachers were given a role in making sure that children needing glasses actually used them in class, this could be a powerful way in increasing the wearing of glasses [24–27]. Regarding children wearing glasses, the glasses worn by 26.05% (81/311) students were not appropriate for vision correction, which may be due to the poor quality of optical shops or a failure to update prescriptions [24–27]. Further research will take measures to overcome hesitancy to wear glasses.

This study showed that the secondary cause of visual impairment was amblyopia. The prevalence of amblyopia was 2.13% in children whose corrected visual acuity in both eyes was less than 20/40. For children with amblyopia refered to the Shanghai Eye Disease Prevention and Treatment Center, we conducted a simple survey about the conscious and treatment of amblyopia, and the results found that 90% of children had not been detected before our study. Therefore, government should take measures to extend amblyopia screening to preschool children in eye health services for children of migrant workers, in case of missing the best treatment time.

This study also showed that 0.08% students suffered from low vision. There was a previous study in schools on the causes of blindness of students with low vision or blindness [28, 29]. Results showed that 20% of the students in shanghai blind children school were children of migrant workers, which indicated that the prevalence of low vision among children of migrant workers was underestimated. During the study, the students with low vision were treated with visual aids, and most of them experienced vision improvement. Efforts were also made to transfer those students to receive Low Vision Rehabilitation in shanghai eye disease treatment and prevention center and special education in shanghai blind children school.

## Conclusions

Our results demonstrated that the refractive error was the principal cause of the visual impairment of the children of migrant workers. The rate of wearing spectacles was low and the percentage of inaccurate prescriptions, among those who wore spectacles, was high. Uncorrected refractive error was a significant cause of visual impairment in migrant children. Attentions should be paid to the problems of visual impairment caused by refractive error. Long-term eye screening programs, primary refractive services and public education should be identified in order to reduce the burden of refractive in children of migrant workers.
